# Identification of Biomarkers in Cerebrospinal Fluid and Serum of Multiple Sclerosis Patients by Immunoproteomics Approach

**DOI:** 10.3390/ijms151223269

**Published:** 2014-12-15

**Authors:** Paolo Colomba, Simona Fontana, Giuseppe Salemi, Marilisa Barranca, Claudia Lo Sicco, Maria Antonietta Mazzola, Paolo Ragonese, Giovanni Savettieri, Giacomo De Leo, Riccardo Alessandro, Giovanni Duro

**Affiliations:** 1 Istituto di Biomedicina e Immunologia Molecolare “A. Monroy”, Consiglio Nazionale delle Ricerche (CNR), Via Ugo La Malfa 153, 90146 Palermo, Italy; E-Mails: paolocolomba@gmail.com (P.C.); riccardo.alessandro@unipa.it (R.A.); giovanni.duro@ibim.cnr.it (G.D.); 2 Dipartimento di Biopatologia e Biotecnologie Mediche e Forensi, Sez. Biologia e Genetica, Università di Palermo, via Divisi 83, 90139 Palermo, Italy; E-Mails: marilisabarranca@yahoo.it (M.B.); claudia.losicco@gmail.com (C.L.S.); giacomo.deleo@unipa.it (G.D.L.); 3 Dipartimento di Biomedicina Sperimentale e Neuroscienze, Università di Palermo, via del Vespro 129, 90127 Palermo, Italy; E-Mails: giuseppe.salemi@unipa.it (G.Sal.); mariaantonietta.mazzola@gmail.com (M.A.M.); paolo.ragonese@unipa.it (P.R.); giovanni.savettieri@unipa.it (G.Sav.)

**Keywords:** multiple sclerosis, cerebrospinal fluid, immunoproteome, anti-transferrin autoantibodies, serum biomarker

## Abstract

Multiple sclerosis (MS) is an autoimmune inflammatory demyelinating disease of the central nervous system. At present, the molecular mechanisms causing the initiation, development and progression of MS are poorly understood, and no reliable proteinaceous disease markers are available. In this study, we used an immunoproteomics approach to identify autoreactive antibodies in the cerebrospinal fluid of MS patients to use as candidate markers with potential diagnostic value. We identified an autoreactive anti-transferrin antibody that may have a potential link with the development and progression of MS. We found this antibody at high levels also in the serum of MS patients and created an immunoenzymatic assay to detect it. Because of the complexity and heterogeneity of multiple sclerosis, it is difficult to find a single marker for all of the processes involved in the origin and progression of the disease, so the development of a panel of biomarkers is desirable, and anti-transferrin antibody could be one of these.

## 1. Introduction

Multiple sclerosis (MS) is one of the most frequent chronic neuroimmunologic disorders of the central nervous system (CNS). It is classified as a multifactorial disease and is characterized by an autoimmune inflammation that results in damage to the myelin sheath and the axons. Several immunopathologic processes have been described as involved in the pathogenesis of MS, such as primary apoptosis of oligodendrocytes [1], dysfunction of regulatory T-cells [2] or B-cell-mediated autoimmunity [3]. These different pathophysiologic processes can selectively predominate in individual patients and contribute to the heterogeneity in the phenotypic expression of the disease, its prognosis and the response to therapies [4]. At present, MS is still diagnosed on a clinical and instrumental basis, and delays in definitive diagnosis are due especially to the lack of diagnostic laboratory tests. Thus, the identification of new biomarkers to measure neurodegeneration could radically change the management of MS and improve diagnostic certainty in the initial phase. Moreover, an early and appropriate diagnosis of MS would allow for more focused therapeutic intervention, thus helping to ensure favorable long-term outcomes.

The immune-mediated etiology of MS has been widely described [5,6], and growing evidence indicates that intrathecal antibody production and the dominance of B-cells are associated with a more progressive course of disease [7]. Currently, detection of oligoclonal Ig is an important diagnostic marker in MS [8,9], though the antigen specificities of these oligoclonal Ig bands has yet to be defined. In recent years, several studies have highlighted the presence of autoantibodies directed against various myelin and non-myelin target antigens in the serum and cerebrospinal fluid (CSF) of MS patients [1,7,10,11,12,13,14,15,16,17]. Some of these studies, as well as others concerning other autoimmune disorders (neuropsychiatric systemic lupus erythematosus and Hashimoto’s encephalopathy) indicate that the immunoproteomics approach is a powerful tool in the field of autoimmunity [15,18,19,20,21].

In this study, we used a proteomic-based analysis to screen for antibodies specifically found in both the CSF and serum of MS patients. Typically, the characterization of autoantibodies in MS patients has been made using purified antigens [10], relevant peptides from preselected targets [22] or a panel of antigens derived from nervous tissue extracts [1,15,21,23]. In this study, for the first time, proteins obtained from CSF were used as a panel of antigens for detecting autoreactive IgG repertoires present in the serum and CSF of MS patients and control subjects. CSF proteins were separated by two-dimensional electrophoresis (2DE) and probed with CSF or serum samples. This immunoproteomics approach allowed us to detect in both the CSF and serum of MS patients autoreactive IgGs that specifically recognize transferrin (Tf) isoforms present in the CSF. In order to propose the anti-transferrin antibodies as putative biomarkers for MS, we focused our attention on serum-autoreactive IgGs, because, as a biomarker source, serum provides several advantages over CSF, including the ease of accessibility and reduced risk to the patient. Thus, we developed an enzyme-linked immunosorbent assay (ELISA) for detecting anti-transferrin antibodies in serum samples. Blind tests performed with our ELISA system on the serum of healthy controls (80), MS patients (124) and patients with other autoimmune and neurologic diseases (39 and 28, respectively) revealed that, by selecting a suitable cut-off, the levels of serum anti-transferrin antibodies can help to discriminate between MS and non-MS subjects.

## 2. Results and Discussion

### 2.1. Identification of Autoreactive IgGs in MS-CSF

In this study, we applied an immunoproteomics approach to evaluate the presence of autoantibodies against antigen targets of CSF in both MS-CSF and MS serum samples.

Since the volume of each MS-CSF sample was highly variable, we decided to pool them and obtained, as described in the Experimental Section, four MS-CSF pools (named MS-CSF Pool I, MS-CSF Pool II, MS-CSF Pool III and MS-CSF Pool IV). In order to obtain a wide repertoire of CSF proteins, the MS-CSF pools were separated by 2DE after depletion of albumin and IgGs. The representative map reported in Figure S1A shows that the depletion of albumin and IgGs does not change the general protein profile of pools that match well with the reference CSF map available in the SWISS 2D-PAGE database (http://world-2dpage.expasy.org/swiss-2dpage/) (Figure S1B).

**Figure 1:**
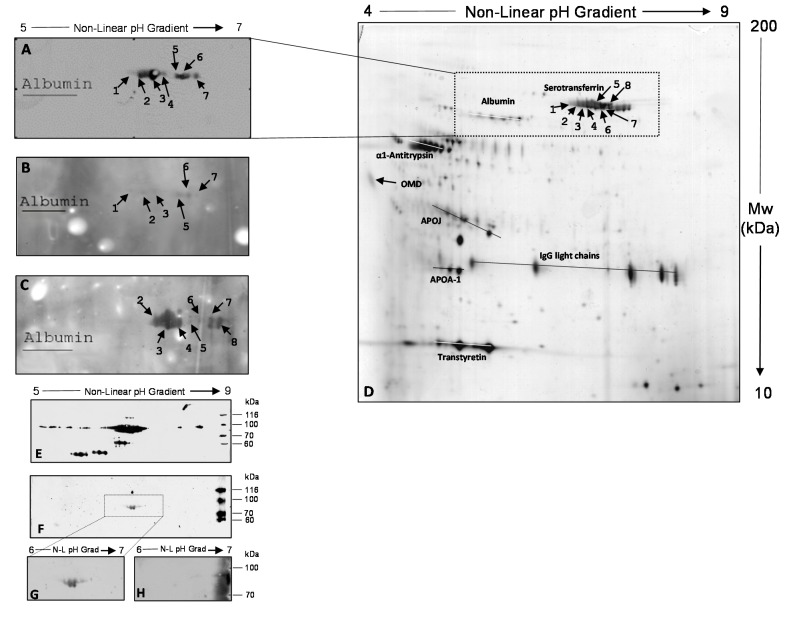
(**A**) 2D-immunoblotting obtained by probing D-MS-CSF Pool I with the corresponding UD-MS-CSF Pool I (UD, undepleted); An equivalent protein pattern was obtained for MS-CSF Pools II, III and IV; (**B**) 2D-immunoblotting obtained by probing D-MS-CSF Pool II with the corresponding UD-MS-CSF Pool II; (**C**) 2D-immunoblotting obtained by probing D-MS-CSF Pool III with the corresponding UD-MS-CSF Pool III; (**D**) silver-stained 2D proteomic map of D-MS-CSF Pool I separated in pH range 4–9. Immunoreactive spots (numbered from 1 to 8) were identified as Tf isoforms by matching immunoblots in (**A**–**C**) with this reference map. The correspondence was established by using albumin isoforms as anchors for matching (see the Experimental Section); (**E**) 2D-immunoblotting obtained by probing Tf purified from human blood with a commercial anti-transferrin antibody; (**F**) 2D-immunoblotting obtained by probing Tf purified from human blood with UD-MS-CSF Pool III; (**G**) Magnification of 2D-immunoblotting reported in (**E**); this image represents the control condition of the competition assay; and (**H**) 2D-immunoblotting obtained by probing purified human Tf with UD-MS-CSF Pool III pre-incubated for five hours with 200 μg/mL of purified Tf. OMD: Orosomucoid-1, synonymous Alpha-1-acid glycoprotein 1; APOJ: Apolipoprotein J, synonymous Clusterin; APOA-1: Apolipoprotein A-I.

Three undepleted MS-CSF pools (UD-MS-CSF Pools I, II and III) were tested with 2D-immunoblotting, using for each the corresponding depleted MS-CSF (D-MS-CSF) pool as the antigenic substrate. As reported in Figure 1A–C, the presence of antigenic spots ranged from pI 6.3 to 6.6, and *M*_W_ from 86 to 77 kDa were detected in all MS-CSF pools. No immunoreactive spot was detected when the three D-MS-CSF pools were probed with three different CSFs obtained from migraine patients used as control.

By using albumin spots as anchors (visible and marked on the nitrocellulose membrane after Ponceau staining), each 2D-immunoblot was matched with the corresponding silver-stained gel (a representative image is reported in Figure 1D), allowing identification of the immunoreactive spots as Tf isoforms.

In order to confirm this identity, we performed a 2D-immunoblotting with UD-MS-CSF Pool III and a commercial anti-transferrin antibody, using Tf purified from human blood plasma as the antigenic substrate. The western blots (WB) in Figure 1E,F show that the spots recognized by antibodies present in UD-MS-CSF Pool III (Figure 1F) correspond to some of the multiple isoforms of Tf recognized by the commercial anti-transferrin antibody (Figure 1E). In order to confirm the specificity of the interaction between autoreactive antibodies and Tf, we performed a competition assay, as described in the Experimental Section. The WB reported in Figure 1H, compared with that in Figure 1G, shows that when UD-MS-CSF Pool III was incubated with 200 µg/mL of purified Tf for five hours, no spot was recognized in MS-CSF, indicating the specificity of CSF autoantibodies/Tf binding. The results indicate that antibodies able to specifically recognize and bind Tf are present in MS-CSF. This binding could cause a decrease in available CSF Tf, with potential consequent effects on its physiologic role in CNS.

Tf is an iron-binding beta-globulin with a weight of 80 kDa and is responsible for most of the cellular iron delivery in the body [24], including the brain [25].

Neurons and glia require iron, as do all cells in the body, for processes of cellular respiration and cell proliferation during development and, additionally, in functions, such as myelination and neurotransmission [26]. Tf is the principal source of iron delivery to the brain and has a pivotal role in maintaining brain iron homeostasis. Even if the mechanism by which Tf enters the brain and delivers iron has been widely described [27,28,29], little is known about its role in iron egress.

Growing evidence suggests that accumulation of iron in the brain contributes to neurodegenerative processes. The cause of this iron overload is not still clearly defined, but may be due to a change in iron delivered via Tf or a decrease in Tf-mediated iron egress from the brain [25]. CSF and serum Tf concentrations in MS patients have been investigated, but with conflicting results [30,31,32,33]. In MS-CSF, rather than a general decrease in Tf levels, the presence of anti-transferrin antibodies could cause a sequestration of Tf, inducing a deficit of available transferrin and the consequent iron homeostasis deregulation observed in the CNS of MS patients.

### 2.2. Identification of Autoreactive IgGs in MS Serum

**Figure 2:**
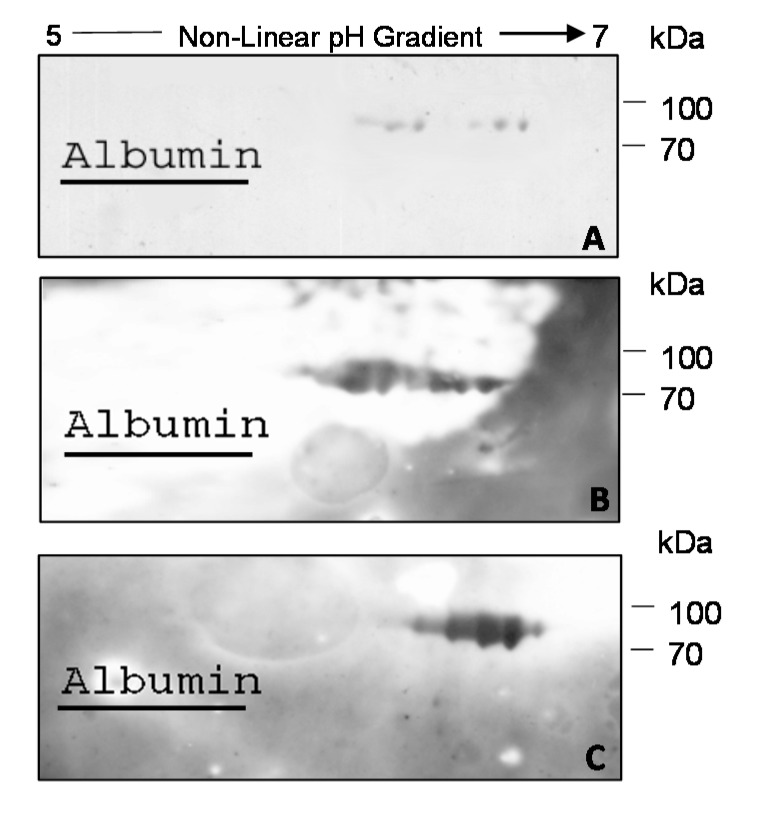
(**A**) 2D-immunoblotting obtained by probing D-MS-CSF Pool IV with the corresponding MS-Se (Se, serum) pool; (**B**) 2D-immunoblotting obtained by probing D-MS-CSF Pool IV with one of the sera used in the MS-Se pool (see Table S1); and (**C**) 2D-immunoblotting obtained by probing Tf purified from human blood with the same serum as in (**B**). Albumin was used as an anchor for matching with the reference CSF map (see the Experimental Section).

In the field of clinical proteomics, one of the major goals is to identify biomarkers from blood samples that could be used to develop non-invasive and cost-effective methods for discriminating between affected and healthy individuals. With this goal, we decided to search for anti-transferrin antibodies also in the serum of MS patients. Thus, we carried out the same assays described above, but using sera samples instead of CSF samples as a source of antibodies. As shown in Figure 2A, the 2D-immunoblotting performed using D-MS-CSF Pool IV as the antigenic substrate allowed the identification of antigenic spots ranging from pI 6.3 to 6.6 and *M*_W_ from 86 to 77 kDa also in the pool of MS sera (MS-Se pool) corresponding to CSF samples of D-MS-CSF Pool IV (see Table S1). The same antigenic spots were visible, even if with different intensity, when D-MS-CSF Pool IV was probed with one of the sera used in the MS-Se pool (see Table S1), indicating that in serum, it is possible to find detectable amounts of these autoreactive IgGs (Figure 2B). Again, in order to confirm that the immunoreactive spots corresponded to Tf isoforms, the single serum used in WB in Figure 2B was assayed with 2D-immunoblotting using the human purified Tf as the antigenic substrate (Figure 2C).

**Figure 3:**
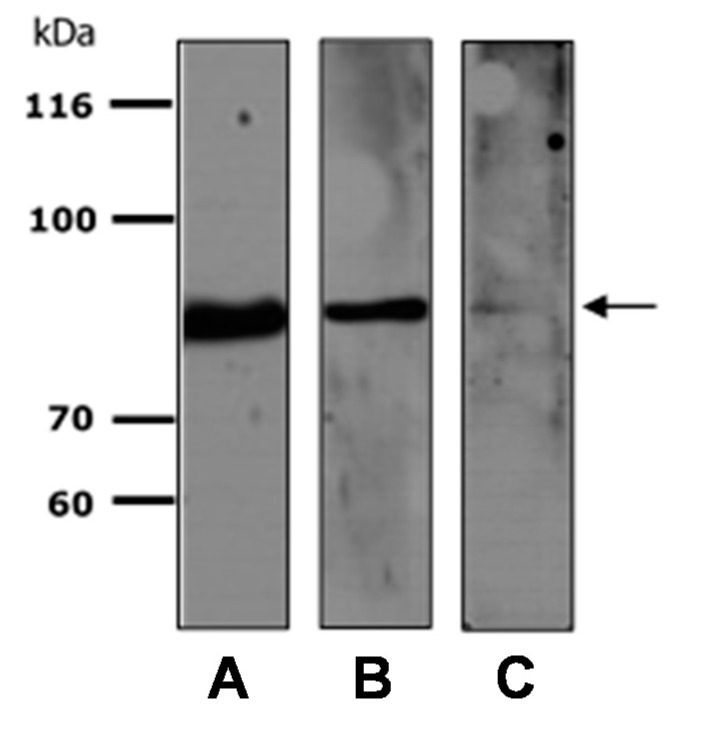
Representative 1D-immunoblotting of recombinant transferrin probed with commercial anti-transferrin antibody (**A**), serum of an MS patient (**B**) and serum of a healthy subject (**C**). The arrow highlights the Tf molecular weight.

Thus, data obtained from the immunoproteomics approach we used show that in both CSF and serum from MS patients, anti-transferrin autoantibodies are detectable. In order to make this detection easier and faster, we tried to assay the presence of these autoantibodies by mono-dimensional immunoblot (1D-immunoblotting) using the recombinant transferrin as the substrate; a simpler technique than 2D-immunoblotting and one that allows the analysis of a greater number of samples. By 1D-immunoblotting, we randomly tested one MS serum and one serum sample from a healthy individual (see Table S1) using the human recombinant Tf as the substrate. The results reported in Figure 3 show that, as well as the commercial anti-transferrin antibody (Figure 3A), the anti-transferrin autoantibodies present in the serum of MS patients are able to recognize the recombinant Tf (Figure 3B). Moreover, we found that anti-transferrin autoantibodies were also present in the serum of healthy individuals (Figure 3C), but in a lower concentration than in MS patients.

The presence of natural autoantibodies (NAAs) in the serum of healthy individuals has been recognized for many years and is widely described in the literature [34,35,36,37], even if their role in the regulation of the immune response and maintenance of immune homeostasis has yet to be clarified. NAAs have been shown to bind to a broad range of evolutionarily conserved cell surfaces and intracellular and circulating antigens, but also self-antigens that are targets of autoantibodies in autoimmune disease (AD), e.g., thyroglobulin, cytoplasmic antigens of polynuclear neutrophils, intrinsic factor, factor VIII, glomerular basement membrane and myelin basic protein ([38] and references therein; [39]). On the basis of current knowledge, it has been concluded that NAAs present in healthy individuals are indistinguishable from the autoantibodies found in AD in terms of V gene usage, extent of mutations, affinity and specific reactivity, but differ in quantity and fine epitope specificity [38,40]. However, the boundaries between physiological autoreactivity and pathological autoimmunity are still ill-defined [41].

The results we obtained from WB analysis, showed that recombinant transferrin is differentially recognized by the autoantibodies present in the serum of healthy individuals and MS patients, probably due to differences in the amount or epitope specificity. In light of this data, we decided to develop, by using the recombinant autoantigen, an ELISA that could potentially offer several advantages: A small amount of required antigen, analysis of multiple serum samples in the same plate and antigen antibody reaction detected by spectrometric analysis [41].

### 2.3. Enzyme-Linked Immunosorbent Assay (ELISA)

**Figure 4:**
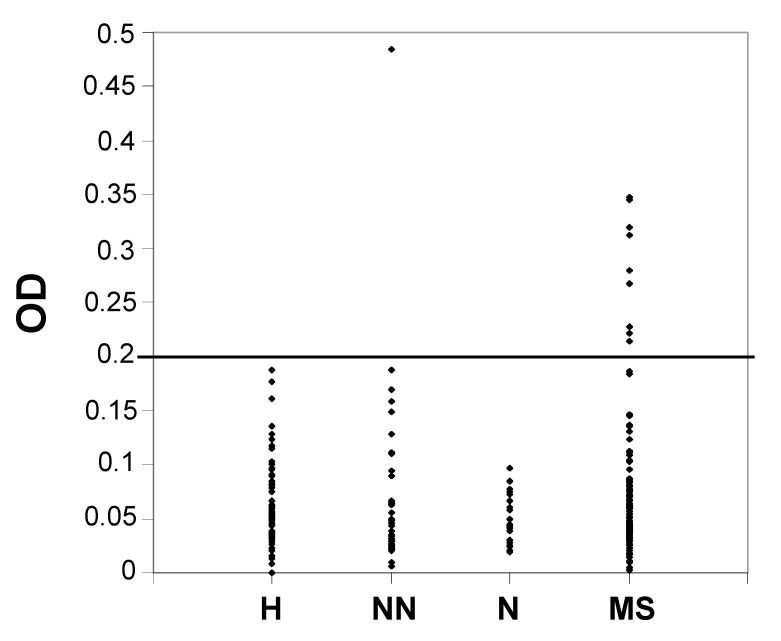
Serum levels of anti-transferrin autoantibodies, assayed with enzyme-linked immunosorbent assay (ELISA), in healthy subjects (H), in patients with non-neurological diseases (NN), in patients with neurological diseases (N) and in MS patients (MS). The cutoff was set at 0.2 OD for the 1:50 dilution.

To determine whether there were significant differences in the serum titers of autoantibodies directed against Tf, we performed an ELISA in four groups of subjects: MS patients, patients with other neurological diseases (some with inflammatory diseases), patients with non-neurological (some with autoimmune diseases) and healthy individuals (see Table S1). Our aim was to investigate the presence of this candidate biomarker in serum specimens and to develop a validated assay to measure its amount. One hundred twenty four sera from MS patients, 28 from patients with neurological diseases, 39 from patients with non-neurological diseases (some of whom had autoimmune diseases) and 80 from healthy individuals (Table S1) were tested with a homemade ELISA. Dilutions of 1:20, 1:50 and 1:100 were assayed for each sample, but the 1:50 dilution provided clearer results, which are reported in the graph in Figure 4. Since no healthy control sample showed an OD above 0.2 (Figure 4 and Table S1), we fixed this value as the cutoff.

We found that nine MS patients had serum levels of anti-transferrin autoantibodies above the cutoff (eight with a relapsing-remitting subtype and one with a secondary-progressive subtype), with no correlation with gender, age, disease duration or the severity of disease, as evaluated with MSSS [42]. The only subject without MS who showed a value above the cutoff was affected with systemic lupus erythematosus, an autoimmune disease that significantly alters the homeostasis of the immune response (Table 1 and Table S1). Thus, we grouped the data obtained into two categories for each class of subjects by using the fixed cutoff at 0.2 OD. A non-parametric χ^2^ analysis was performed showing a statistically significant difference between the MS group and the control group (*p* = 0.0045). Further comparisons between the MS group and one of the three subgroups of controls showed that a statistically significant difference persisted when we compared the MS group with the healthy control group (*p* = 0.01), while no statistically significant difference was observed when we compared the MS group with the group with no neurological disease (*p* = 0.26) or with the neurological diseases group (*p* = 0.151), perhaps due to the low number of these two subgroups. Moreover, by using the same fixed cutoff at 0.2 OD, the sensitivity of the assay was 7.3%, the specificity was 99.3%, the positive predictive value was 90.0% and the negative predictive value was 55.9%. Because of the high specificity, we can say that serum anti-transferrin antibodies could be used as biomarkers for identifying MS patients when the value of the ELISA test is above 0.2 OD. On the other hand, the low sensitivity observed should not be surprising, since it is well known that a single marker is unlikely to serve as a general diagnostic or prognostic tool for covering the heterogeneity that often characterizes a pathological condition. Therefore, the development of a panel of biomarkers, specific for different pathophysiologic mechanisms, should be considered more appropriate for further understanding the pathogenesis of MS, as well as for diagnosis, classification, evaluation of disease activity and theranostic applications [21,43].

**Table 1:** Results of the ELISA assay.

OD ^a^	MS patients	WO MS ^b^	Total
≥0.2	9	1	10
<0.2	115	146	261
Total	124	147	271

^a^ Optical density; ^b^ Individuals without MS. This group includes both healthy subjects and patients with non-neurologic and neurologic diseases.

## 3. Experimental Section

### 3.1. Chemicals

All chemicals, where not otherwise indicated, were purchased from Sigma–Aldrich, St Louis, MO, USA.

### 3.2. Cerebrospinal Fluid (CSF) and Serum Samples

Sera and CSF were obtained for diagnostic purposes and after written informed consent. Twenty seven CSF samples obtained from MS patients by lumbar puncture were provided by the Department of Experimental Biomedicine and Clinical Neurosciences, University of Palermo, Palermo, Italy. All MS patients, diagnosed according to the McDonald criteria, were affected with relapsing-remitting MS (RR-MS) and were used to obtain 4 pools (MS-CSF pools). These pools were obtained by mixing aliquots of 800 μL taken from each CSF sample. As reported in Table S1, MS-CSF Pool I was composed of 3 samples, MS-CSF Pool II of 7 samples, MS-CSF Pool III of 9 samples and MS-CSF Pool IV of 8 samples. Three CSF samples from migraine patients who showed normal CSF biological parameters were used as controls (ctr-CSF). CSF samples (both single and pooled) were centrifuged at 1000× *g* (4 °C) for 10 min to eliminate cells and other insoluble material; the supernatants were dialyzed to remove salt, lyophilized to concentrate and then stored at −80 °C until further processing. Protein concentration was determined according to the Bradford method [44].

One hundred thirty one serum samples from patients with MS (MS-Se), 27 of whom were counterparts of CSF previously described, 28 from patients with various neurologic diseases (NEU-Se), and 80 from healthy individuals (H-Se) were also provided by the Department of Experimental Biomedicine and Clinical Neurosciences, University of Palermo. Among the MS-Se samples, 73 were obtained from patients with RR-MS, 13 from patients with primary progressive multiple sclerosis (PP-MS), 41 from patients with secondary progressive multiple sclerosis (SP-MS) and 4 from patients with progressive relapsing (PR-MS). Moreover, 39 serum samples from patients with non-neurologic diseases, some of whom had autoimmune diseases, (NoNEU-Se), were provided by the Department of Internal and Specialist Medicine, University of Palermo. All of the information on CSF and serum samples used in this study is reported in Table S1. All serum samples were obtained by venous blood collection and were collected in plain tubes, allowed to clot for 1 h and centrifuged twice at 2500× *g* (4 °C) for 10 min to obtain serum. Each serum sample was aliquoted and stored at −80 °C until further processing.

### 3.3. Albumin and IgG Depletion from MS-CSF Pools

To increase the sensitivity and the resolution of the immunoproteomics approach we applied in this study, an aliquot of MS-CSF Pools I, II, III and IV was albumin- and IgG-depleted using the Albumin and IgG Depletion SpinTrap kit (GE Healthcare, Piscataway, NJ, USA) following the manufacturer’s instructions. These depleted MS-CSF Pools (D-MS-CSF Pools) were subjected to high-resolution 2DE and used as the source of auto-reactive CSF proteins. The undepleted aliquots of MS-CSF pools (UD-MS-CSF pools) were used, together with serum samples, as the source of auto-antibodies in the immunoproteomics assays described in the next paragraph.

### 3.4. Two-Dimensional Electrophoresis (2DE), 2D-Immunoblotting and Competition Assay

Lyophilized MS-CSF proteins and purified transferrin were solubilized in a buffer containing 8 M urea, 4% *w*/*v* CHAPS (3-[(3-cholamidopropyl)dimethylammonio]-1-propanesulfonate), 40 mM Tris, 65 mM DTE (1,4-Dithioerythritol) and a trace amount of bromophenol blue and subjected to high resolution 2DE, as previously reported [45,46]. Isoelectrofocusing was done on a non-linear immobilized pH gradient (pH 3–10; 18 cm-long IPG strips; GE Healthcare), and second dimension separation was done on 9%–16% SDS-polyacrylamide gradient gels. For each kind of sample, a reference gel stained with ammoniacal silver staining was prepared.

For the 2D-immunoblotting, gels were blotted onto nitrocellulose membranes (Hybond-ECL, GE Healthcare) in transfer buffer containing 25 mM Tris, 192 mM glycine and 20% methanol. After blotting, the membranes were stained with Pounceau red and scanned to subsequently use the visible spots as the anchor for matching with the reference 2D gel and autoradiographs. Then, the membrane was abundantly washed with water to remove the Pounceau red, blocked with 5% BSA (Bovine Serum Albumin) in TBST (20 mM Tris, 140 mM NaCl, 0.1% Tween-20) for 1 h and incubated overnight with sera (1:10,000) or undepleted CSF pools (1:100). After washing, membranes were incubated with anti-human IgG horseradish peroxidase (HRP) conjugate (1:20,000; GE Healthcare). Alternatively, membranes in which purified Tf was blotted were incubated with a polyclonal anti-transferrin antibody (1:1000; H-65, Santa Cruz Biotechnology, Santa Cruz, CA, USA). After incubation with the appropriate secondary HRP-conjugated Ig, the signal was developed with the enhanced chemiluminescence detection system (Super Signal, Thermo Scientific, Rockford, IL, USA). The reference silver-stained 2D gel, the stained membranes and the autoradiographs were matched using the Image Master 2D Platinum v6.0 software to analyze the immune profiles (GE Healthcare).

The competition assay was done by pre-incubating UD-MS-CSF Pool IV (1:100 in TBS-T) for 5 h with 200 µg/mL of Tf purified from human plasma. After incubation, the mixture was used to probe a membrane in which the 30 µg of purified transferrin had been blotted.

### 3.5. 1D-Immunoblotting

About 0.5 μg of recombinant Tf was subjected to SDS-PAGE in 8% polyacrylamide gel and transferred to a nitrocellulose membrane. The membrane was incubated in blocking solution (5% non-fat dry milk in TBST) and probed overnight at 4 °C with the serum from 1 MS patient and from 1 healthy control randomly selected, diluted at 1:10,000. In addition, a polyclonal antibody against Tf (H-65), diluted at 1:1000, was used as a control to detect the antigen. After several washings, the membranes were incubated with horseradish peroxidase-conjugated secondary antibody anti-human-IgG or anti-rabbit-IgG, diluted at 1:20,000 and 1:10,000, respectively, and proteins detected by the enhanced chemiluminescence detection system.

### 3.6. ELISA Assay

The amount of anti-transferrin antibody in serum specimens was measured with ELISA. One hundred twenty four samples from MS patients, 28 from patients with neurologic disorders, 39 from patients with non-neurologic diseases and 80 from healthy individuals were assayed (Table S1).

The antigen was pre-coated onto a polystyrene microplate (96-well Flat Bottom, High Bind, Easy Wash Plate, Corning, NY, USA): 50 μL/well of recombinant transferrin diluted at 3.2 μg/mL in sodium carbonate/bicarbonate buffer 0.1 M, pH 9.4, was incubated overnight at 4 °C. The plate was then washed with 300 μL of PBS-T (137 mM NaCl, 2.7 mM KCl, 10 mM Na_2_HPO_4_, 2 M KH_2_PO_4_, pH 7.4, with 0.05% Tween-20) and blocked for 1 h with 300 μL of 2% BSA in PBS-T. After washing with PBS-T, 50 μL/well of serial dilutions 1:20, 1:50 and 1:100 of serum samples from patients and control subjects in 2% BSA in PBST were incubated for 2 h at room temperature in the pre-coated microplate; any anti-transferrin antibody present was bound by the immobilized antigen. After several washings in PBST to remove any unspecific antibodies, 50 μL of rabbit anti-human IgG/HRP conjugate (DakoCytomation, Carpinteria, CA, USA) diluted 1:5000 in 2% BSA in PBS-T were added to each well and incubated 1 h at room temperature. After several washings in PBS-T to remove any unbound anti-IgG/enzyme reagent, 100 μL of substrate TMB (3,3',5,5'-Tetramethylbenzidine; Thermo Scientific) was added to each well, and after 15–20 min of incubation, the color development was stopped with an equal volume of 2 M H_2_SO_4_. The intensity of the color was measured by absorbance at 450 nm using a microtiter plate reader (Biotek, Winnoski, VT, USA); color development was in proportion to the amount of anti-transferrin antibody that was bound in the initial step. As a blank control, the antibody anti-human IgG/HRP conjugate diluted in 2% BSA in PBST was used. No significant cross-reactivity or interference was observed.

Statistical analysis of the data was carried out with the χ^2^ at a significance level of 0.05.

## 4. Conclusions

This study is the first to use an immunoproteomics approach to identify human CSF proteins that induce specific antibody responses in MS patients, leading to the discovery of anti-transferrin autoantibodies as a new candidate biomarker. The detection of these autoantibodies not only in CSF, but also in sera samples of MS patients prompted us to develop an unbiased, non-invasive and fast method based on ELISA testing. The results show that the evaluation of serum anti-transferrin autoantibodies levels helps in discriminating individuals with MS from healthy ones. However, the impossibility of finding a single antibody that fulfills all of the criteria of a surrogate endpoint in MS is well known. Thus, rather than as a single biomarker, the anti-transferrin autoantibodies could be used in combination with other antibodies already described as putative MS biomarkers [7,15,16,21] to create a panel consonant with the complexity of the disease that can aid in optimizing diagnosis, therapy and preventing disability.

## Supplementary Materials

Supplementary materials can be found at http://www.mdpi.com/1422-0067/15/12/23269/s1.
